# Understanding the Intrinsic Carrier Transport in Highly Oriented Poly(3-hexylthiophene): Effect of Side Chain Regioregularity

**DOI:** 10.3390/polym10080815

**Published:** 2018-07-25

**Authors:** Sanyin Qu, Chen Ming, Qin Yao, Wanheng Lu, Kaiyang Zeng, Wei Shi, Xun Shi, Ctirad Uher, Lidong Chen

**Affiliations:** 1 State Key Laboratory of High Performance Ceramics and Superfine Microstructure and CAS Key Laboratory of Materials for Energy Conversion, Shanghai Institute of Ceramics, Chinese Academy of Sciences, Shanghai 200050, China; qusanyin@mail.sic.ac.cn (S.Q.); mingchen@mail.sic.ac.cn (C.M.); shiwei@student.sic.ac.cn (W.S.); xshi@mail.sic.ac.cn (X.S.); 2 Department of Mechanical Engineering, National University of Singapore, Singapore 117576, Singapore; luwanheng@u.nus.edu (W.L.); mpezk@nus.edu.sg (K.Z.); 3 University of Chinese Academy of Sciences, Beijing 100049, China; 4 Department of Physics, University of Michigan, Ann Arbor, MI 48109, USA; cuher@umich.edu; 5 Shanghai Institute of Materials Genome, Shanghai 200050, China

**Keywords:** organic thermoelectric, poly(3-hexylthiophene), regioregularity, carrier transport

## Abstract

The fundamental understanding of the influence of molecular structure on the carrier transport properties in the field of organic thermoelectrics (OTEs) is a big challenge since the carrier transport behavior in conducting polymers reveals average properties contributed from all carrier transport channels, including those through intra-chain, inter-chain, inter-grain, and hopping between disordered localized sites. Here, combining molecular dynamics simulations and experiments, we investigated the carrier transport properties of doped highly oriented poly(3-hexylthiophene) (P3HT) films with different side-chain regioregularity. It is demonstrated that the substitution of side chains can not only take effect on the carrier transport edge, but also on the dimensionality of the transport paths and as a result, on the carrier mobility. Conductive atomic force microscopy (C-AFM) study as well as temperature-dependent measurements of the electrical conductivity clearly showed ordered local current paths in the regular side chain P3HT films, while random paths prevailed in the irregular sample. Regular side chain substitution can be activated more easily and favors one-dimensional transport along the backbone chain direction, while the irregular sample presents the three-dimensional electron hopping behavior. As a consequence, the regular side chain P3HT samples demonstrated high carrier mobility of 2.9 ± 0.3 cm^2^/V·s, which is more than one order of magnitude higher than that in irregular side chain P3HT films, resulting in a maximum thermoelectric (TE) power factor of 39.1 ± 2.5 μW/mK^2^ at room temperature. These findings would formulate design rules for organic semiconductors based on these complex systems, and especially assist in the design of high performance OTE polymers.

## 1. Introduction

Organic materials have attracted increased attention in thermoelectric (TE) research due to their properties of light weight, low cost, and flexibility. Conjugated polymers are intensively studied as organic TE materials and generally expected to be advantageous for enhancing TE properties in terms of good electrical conductivity [1,2,3,4]. In doped conjugated polymer TE materials, the electrical carriers are acknowledged as polarons and bipolarons, which are caused by intramolecular geometric distortions through doping [5]. Carrier transport behaviors, including the transport edge, the dimensionality of the transport path, and carrier mobility, have been identified as the most critical factors for the performance of organic TE materials [6]. The underlying parameters, which may determine the carrier transport properties, include the molecular/electronic structure as well as the packing and/or alignment of polymer chains. Understanding the effect of intrinsic molecular structure and arrangement of polymer chains in the carrier transport behavior of doped conjugated polymer TE materials can not only draw a clear picture of the carrier transport process, but also may assist in the design of high performance TE polymers.

The majority of conjugated polymer TE materials reported to date are traditional highly conducting polymers and some newly designed molecular structures. The traditional conducting polymers such as poly(3,4-ethylenedioxythiophene) (PEDOT) and polyaniline (PANI) have been reported to exhibit high TE performance [7,8]. However, due to the processing conditions as well as the poor solubility of these polymers, the tuning of the molecular structure of these polymers are limited. For the newly designed molecular structures [9,10] generally inspired by organic semiconductors such as organic light-emitting diodes (OLEDs), organic photovoltaics (OPVs), and organic thin-film transistors (OTFTs), these are still limited by their critical processing conditions and uncontrollable molecular weight, as well as unpredictable molecular arrangement. Poly(3-hexylthiophene) (P3HT) is a type of conjugated polymer with easy processing, tunable molecular weight, and molecular structure, which can be served as an idea model system for understanding the correlation between the molecular structure and carrier transport behavior in organic TE materials [11,12,13]. In P3HT, the different attachment positions of side chains on the thiophene group can lead to the formation of a large family of bond-isomers with different configurations, such as head-to-head (HH), head-to-tail (HT), and tail-to-tail (TT) side chain attachments, which are also referred to as the regioregularity (RR) [14]. Recently, it was found that the RR of pristine P3HT thin films are strongly correlated with charge transport properties in OPVs and OTFTs [15,16]. However, there are no precise studies on the effect of the RR on the carrier transport properties of doped P3HT, and therefore on the OTE performance is still kept unknown.

Generally, P3HT thin films are fabricated by self-assembly approaches [17,18]. The large free energy barriers to crystallization lead to great structural inhomogeneity. Therefore, carrier transport behavior in such a system presents average properties contributed by the carrier transport through multiple channels, such as intra-chain, inter-chain, and inter-grain, as well as hopping between disordered localized sites [19]. This makes it challenging to understand the intrinsic effect of side chain RR on carrier transport properties of these conjugated polymer systems.

In our previous studies, we obtained highly oriented P3HT films through the combination of organic small-molecule epitaxy method using 1,3,5-trichlorobenzene (TCB) as the template and a temperature gradient assisting the crystallization process [20]. This allowed us to study the macroscopic carrier transport properties of quasi-one-dimensional P3HT films along or across the backbone chain orientation by suppressing or diminishing the effects of structural disorder in the polymer chains. Therefore, this provided us a possible way to understand the relationship between molecular structure (such as side chain RR) and the carrier transport properties of doped quasi-one-dimensional P3HT films.

In this study, a combination of molecular dynamics simulations and experimental investigations was performed to understand the side chain effect on the carrier transport properties in the doped highly oriented P3HT films. Two such films were prepared through the small molecule epitaxy method using two typical P3HT polymers with different side chain regularity (a regular sample containing >98% of HT attached side chain dyads and an irregular sample containing ~50% of HT attached side chain dyads). It was revealed that the regular substitution of side chain led to a smaller dihedral torsion angle between the thiophene rings, which not only reduced the carrier transport edge, but also allows for one-dimensional transport in the film, and thereby significantly improve the carrier mobility and TE performance. These findings may assist in the design of high performance TE polymers.

## 2. Experimental Procedures

### 2.1. Raw Materials

The P3HT powder samples (rg-P3HT:HT-P3HT, the percentage of molecules with the head-to-tail configuration is up to 98%; ra-P3HT: the ratio of molecules with the head-to-tail and head-to-head configuration HT-HT:HH-HH = 1:1) were purchased from Sigma-Aldrich (Shanghai, China) and used as received (The macromolecular parameters are shown in Table S2). 1,3,5-trichlorobenzene (TCB) was purchased from TCI (Shanghai, China). Trifluoromethanesulfonimide (CF_3_SO_2_)_2_NH (95%) was purchased from Aladdin (Shanghai, China). Ferric sulfate Fe_2_(SO_4_)_3_, sodium bicarbonate NaHCO_3_, and toluene were used as received from Sinopharm Chemical Reagent Company (Shanghai, China). Nitromethane and acetonitrile of analytical grade were dried over calcium chloride CaCl_2_ and phosphorus pentoxide P_2_O_5_ before use, respectively.

### 2.2. Preparation of Oriented ra-P3HT-TCB and rg-P3HT-TCB by TCB Epitaxy

**Figure 1:**
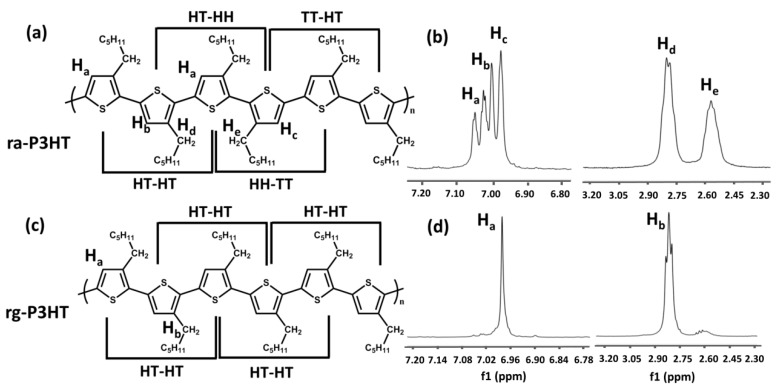
(**a**) Molecular structure of random poly(3-hexylthiophene) (ra-P3HT) with HT-HT:HT-HH:TT-HT:HH-TT=1:1:1:1; (**b**) 1H-NMR spectra of random P3HT with characteristic peaks of protons in thiophene rings in chemical shifts near *δ* = 7.0 ppm, and characteristic peaks of protons in α-methylene protons of hexyl group in chemical shifts near *δ* = 2.4–2.8 ppm; (**c**) Molecular structure of regular P3HT (rg-P3HT) with HT-HT-only structure; (**d**) 1H-NMR spectra of regular P3HT with characteristic peaks of protons in thiophene rings in chemical shifts near *δ* = 7.0 ppm, and characteristic peaks of protons in α-methylene protons of hexyl group in chemical shifts near *δ* = 2.4–2.8 ppm.

The preparation method followed the literature [21]. First, ra-P3HT and rg-P3HT powders were dissolved in toluene to make a 0.5 wt % P3HT solution and then drop-casted on 1.8 mm × 1.8 mm clean glass substrates at 40 °C. Next, 60 mg of the TCB powder was uniformly deposited onto the surface of the ra-P3HT and rg-P3HT films and sandwiched between the P3HT-coated glass substrate and a clean glass coverslip. The ‘sandwich’ was then heated to the melting point of TCB (75 °C). Following heating, the ‘sandwich’ was slowly moved onto another bench at room temperature (25 °C). The TCB was crystallized uniformly along the temperature gradient in ~1 s, and P3HT was crystallized on the TCB microcrystals. After removing the coverslip, the film was further doped by immersion into a solution of the dopant Fetf (Fe(TFSI)_3_) in nitromethane, which was synthesized by treating freshly prepared Fe(OH)_3_ with the acid (CF_3_SO_2_)_2_NH in anhydrous nitromethane. After doping for 1.5 h, the film was rinsed with anhydrous nitromethane and dried under vacuum at room temperature. Finally, the TCB was removed by dissolution in the nitromethane solution. The thickness of the final film was ~2 μm. The obtained product was denoted as ra-P3HT-TCB and rg-P3HT-TCB, respectively (as shown in Figure 1).

### 2.3. Characterization of Microstructure and Electrical Transport Properties

Proton nuclear magnetic resonance (NMR) spectra were obtained using a Bruker AM 500 spectrometer (Bruker, Switzerland), relative to tetramethylsilane (TMS). The absorption spectra of the films were measured using a Varian Cary 500 spectrophotometer (Shanghai, China). The morphologies of the films were characterized by scanning electron microscopy (SEM, FEI Magellan 400 and Zeiss Supra 55). Grazing-incidence X-ray diffraction (GIXRD) data were collected using a D8 Discover Davinci equipped with CuKa radiation (=0.15406 nm). The electrical conductivity was measured using a HL 5500 PC Hall-effect measurement system (Shanghai, China) by the four probe method on a square film sample at room temperature. The film thickness was measured by a profilometer (DEKTAK-150) (Veeco, New York, NY, USA). The carrier concentration, carrier mobility and temperature dependent electrical resistivity measurements were performed in a quantum design physical property measurement system (PPMS, Quantum Design, Beijing, China) by the hall effect method. The Seebeck coefficient was measured from the slope of the produced TE voltage as a function of the temperature difference along the length of the sample. The power factor was calculated from the corresponding Seebeck coefficient and electrical conductivity at room temperature. C-AFM measurements were conducted on a commercial scanning probe microscopy (SPM) instrument (MPF-3D, Asylum Research, Santa Barbara, CA, USA) with a commercial conductive AFM module (ORCA, Asylum Research, Santa Barbara, CA, USA). A PtSi conductive tip (Nanosensors, Neuchatel, Switzerland) was used and the measurements were done with a fixed sample bias of 0.1 V or 0.5 V, and a scan rate of 0.5 Hz. The cantilever has the resonance frequency about 75 kHz, a spring constant of 2.8 N m^−1^, and a tip radius of ~20 nm. All measurements were performed at room temperature and ambient conditions. The topography and current images were obtained simultaneously during the scan. For each sample, at least three different locations were imaged for the C-AFM measurements. In this work, we conducted the current measurements on the in-plane surface, which was consistent with the direction of the carrier transport parameter measurements. To get the in-plane current, the electrode and the C-AFM tip were fixed at the surface and edge of the sample; this configuration gives several millimeters distance between the conductive tip and the electrode for wiring.

### 2.4. Molecular Dynamics Simulation

The force field by Bhata et al. [22] for P3HT was adopted in the simulation. A cut-off distance of 11 Å was used for the non-bonded interactions and a long-range van der Waals tail correction was added to the energy. A particle-particle/particle-mesh Ewald algorithm was used to calculate the long range Coulomb interaction. The velocity-Verlet integrator was used with a time step of 1 fs for integration. The Nosé-Hoover style thermostat and barostat were used to keep the constant temperature and pressure of the system with a damping time of 100 fs and 1 ps, respectively. The structures were relaxed for 4 ns to reach the equilibrium and another 4 ns to gather the data. All the simulations were implemented within the LAMMPS simulation package [23].

## 3. Results and Discussion

### 3.1. Molecular Structure of P3HT with Different Regioregularity

Figure 1 illustrates the chemical structures of regioregular P3HT (rg-P3HT) and regiorandom P3HT (ra-P3HT). Both the rg-P3HT and ra-P3HT materials were purchased from Sigma-Aldrich (Shanghai, China) with molecular weight (*M*w) of 87 kg/mol and used as received. During the synthesis process of P3HT, coupling of 3-substituted thiophenes via the 2- and 5-positions led to the formation of head-to-head (HH), head-to-tail (HT), and tail-to-tail (TT) configurational isomers, which resulted in various degrees of RR of the P3HT structure. As shown in Figure 1a, ra-P3HT consisted of random HT-HT, TT-HT, HT-HH, and TT-HH attachments, while rg-P3HT in Figure 1c consisted of only HT-HT dyads with completely symmetrical structure. The ^1^H-NMR spectrum of ra-P3HT showed four chemical shifts of the distinct protons on the thiophene ring, confirming the random HT-HT(*δ* = 6.98)/TT-HT(7.00)/HT-HH (7.02)/TT-HH (7.05) linkages of the chain. The content of the HT linkage at *δ* = 2.8, estimated by the spectrum analysis, was about 50% in ra-P3HT. On the other hand, for rg-P3HT, only one sharp band corresponding to the thiophene proton at *δ* = 6.98 is observed in the ^1^H-NMR spectrum, which denotes the HT-HT structure. The *α*-methylene proton region showed mostly a triple peak at *δ* = 2.80. The estimated HT linkage in rg-P3HT was more than 98%.

**Figure 2:**
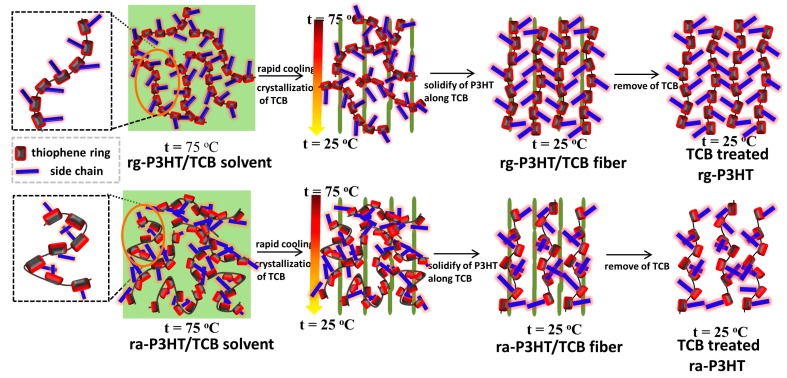
A schematic diagram of preparation of oriented rg-P3HT-TCB and ra-P3HT-TCB with fiber-like texture through a 1,3,5-trichlorobenzene (TCB) molecule epitaxy process. The mixture of rg-P3HT/TCB and ra-P3HT/TCB becomes a solvent after heating to 75 °C, and then the mixture cools down rapidly to 25 °C from one side to the other, forming a large temperature gradient. TCB solidified as a needle-like crystal along the temperature gradient. After doping and removing TCB, both of the rg-P3HT and ra-P3HT polymer chains formed a highly oriented structure.

Figure 2 shows a schematic illustration of the synthesis procedure of oriented ra-P3HT and rg-P3HT films by TCB induced epitaxy. Both ra-P3HT and rg-P3HT were prepared in the form of sandwiched films through drop-casting P3HT on a glass substrate, depositing TCB onto the surface of the drop-casted P3HT film, and finally covering the P3HT/TCB film with a P3HT-coated glass substrate. The sandwiched ra-P3HT/TCB/ra-P3HT and rg-P3HT/TCB/rg-P3HT were heated to the melting point (75 °C) of TCB and both mixtures became solvent. Then, by sliding slowly and smoothly the “sandwiches” from the hot plate (75 °C) onto a cold bench (25 °C), the TCB crystallized uniformly along the temperature gradient and ra-P3HT and rg-P3HT were crystallized onto the TCB templates (microcrystals). Finally, the oriented films were further doped by immersion into a solution of the dopant Fetf (Fe(TFSI)_3_) in nitromethane, and the TCB was removed at the same time. The obtained films were denoted as ra-P3HT-TCB and rg-P3HT-TCB, respectively (as shown in Figure 2). The self-assembled ra-P3HT and rg-P3HT films doped with Fetf are also prepared for comparison. The doping process was verified by ultraviolet-visual light-near-infrared (UV-vis-NIR) absorption spectra as shown in Figure S1.

**Figure 3:**
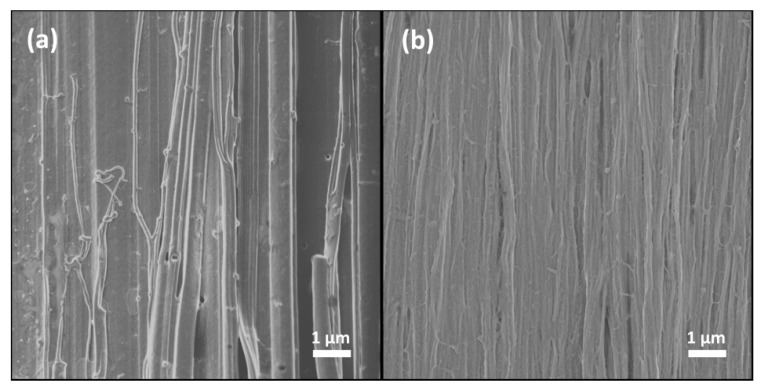
Scanning electron microscopy (SEM) images of (**a**) oriented ra-P3HT-TCB and (**b**) rg-P3HT-TCB films. Both samples show fiber structure in almost only one direction. The ra-P3HT-TCB film has fibers with the diameter from 100 nm to 500 nm. The rg-P3HT-TCB film shows a more uniform distribution of fibers with the diameter of 100–200 nm.

**Figure 4:**
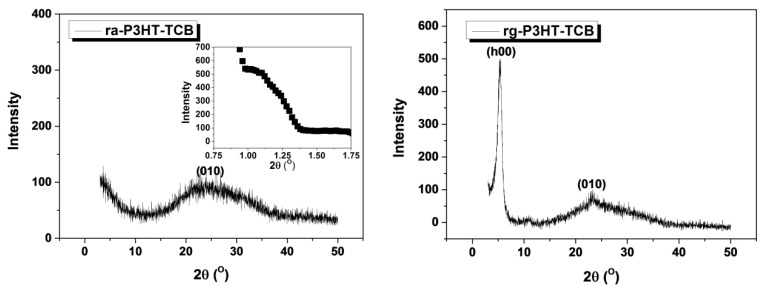
XRD patterns of ra-P3HT-TCB and rg-P3HT-TCB.

Figure 3 depicts the scanning electron miscroscopy (SEM) images of ra-P3HT-TCB and rg-P3HT-TCB. As shown in the figure, both films presented a dense fiber-like structure with different fiber-bundle diameters. The ra-P3HT-TCB sample had a relatively wide distribution of the fiber diameter from 100 nm to 500 nm, while the rg-P3HT-TCB showed a more uniform distribution, with fiber diameters ranging between 100–200 nm. The SEM images of ra-P3HT and rg-P3HT films are shown in Figure S2. Grazing incidence X-ray diffraction was performed to characterize the molecular chain arrangement in the ra-P3HT-TCB and rg-P3HT-TCB films, as shown in Figure 4. The (h00) peak refers to the repeat distance of thiophene rings in the direction of alkyl stacking (also described as an ‘edge on’ arrangement), and (0k0) refers to the repeat distance of thiophene rings in the direction of π-π stacking (also described as a ‘face on’ arrangement). In the ra-P3HT-TCB, a wide range peak at around 2*θ* = 22° was observed, which was ascribed to the (010) peak, indicating that some part of the ra-P3HT-TCB film has the ‘face-on’ periodic arrays of thiophene. However, the (h00) peak in the spectra was missing. We extended XRD measurements to the small angle region to check on the existence of the (h00) peak. As shown in the inset of Figure 4a, there only exists a quite broad peak at around 2*θ* = 1.2°. The results indicate that the arrangement in the direction of alkyl stacking lacked periodicity in the ra-P3HT-TCB film. On the other hand, for the rg-P3HT-TCB film, a strong and sharp peak was observed at 2*θ* = 5.3°, which was assigned to lattice planes (h00). The d-spacing of the (h00) peak was calculated to be ~16.7 Å. The (010) peak, which appeared at 2*θ* = 24.2° with d-spacing of ~3.7 Å, was also observed in the rg-P3HT-TCB film, revealing a sharper peak than in the ra-P3HT-TCB film. The XRD spectrum of rg-P3HT-TCB indicates that the film has both ‘edge-on’ and ‘face-on’ periodic arrays of thiophene. Besides, the stronger and sharper diffraction peaks in rg-P3HT-TCB indicated a more orderly chain arrangement in rg-P3HT-TCB than in ra-P3HT-TCB.

### 3.2. Molecular Dynamics Simulation of Close-Packing Structures of rg- and ra-P3HT

**Figure 5:**
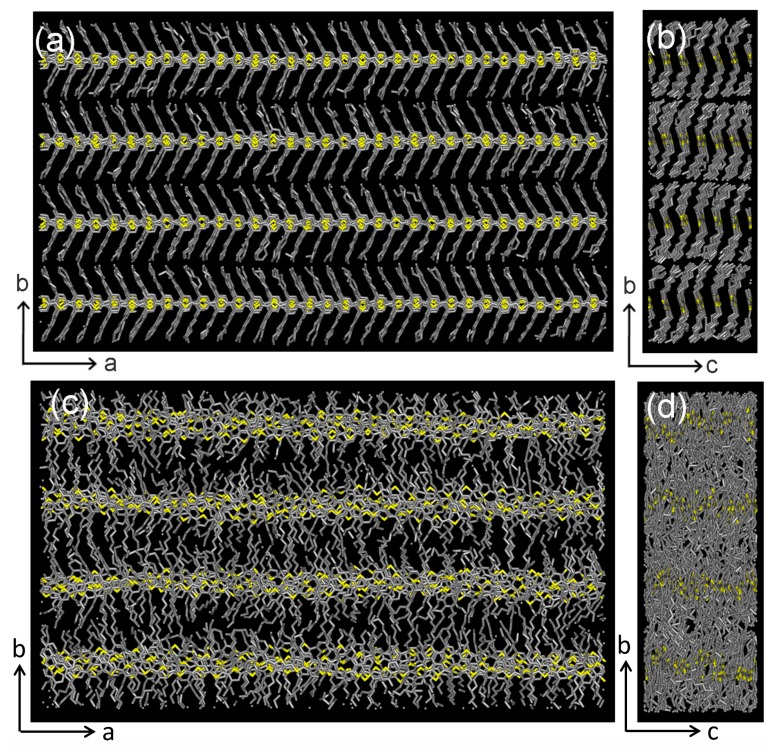
Top view (**a**) and side view (**b**) of the simulation snapshots for rg-P3HT chains; top view (**c**) and side view (**d**) of the simulation snapshots for ra-P3HT chains. The hydrogen atoms are not shown.

To gain further direct insight into the exact molecular packing structures, we modeled the close-packing structures of rg- and ra-P3HT by molecular dynamics (MD) simulations. For rg-P3HT, the non-interdigitated crystalline structure was used as the initial structure for our simulation because it yields the lattice parameters and density close to experimental values [22,24]. To model ra-P3HT, the HT linkages in rg-P3HT were randomly replaced by HH and TT ones to reach the equal content of HT, HH, and TT configurations to mimic the experimental sample. The operating unit used in the simulations consists of 24 chains, with six chains packing along the *c* direction (π-π stacking) and four chains aligning along the b direction. Each chain contains 32 thiophene rings along the *a* direction, as depicted in Figure 5. The periodic boundary conditions were used to simulate infinite chains in order to approach the experimental samples with high molecular weight. The structures were equilibrated via an isothermal-isobaric ensemble at 300 K and a pressure of 1 atm.

The equilibrated structures of rg-P3HT and ra-P3HT are shown in Figure 5. The film of rg-P3HT has a quite ordered packing structure, which yields an average π-π stacking distance of 3.77 Å along the c direction and an aligning distance of 17.92 Å.

Along the b direction, in quite a good agreement with the values of 3.7 and 16.7 Å obtained from XRD measurements. In contrast to rg-P3HT, the ra-P3HT film shows a much less ordered packing not only along the b direction (Figure 5c), but also along the π-π stacking direction (c direction) (Figure 5d). This feature originated from the steric hindrance interactions between the disordered side chains, which significantly breaks the planarity of the backbones and affects the order of inter-chain π-π stacking between the thiophene rings. The less-ordered π-π stacking feature in the ra-P3HT film obtained from the simulation agreed well with the XRD measurement as the corresponding peak was not as sharp as that in the rg-P3HT film (see Figure 4). The average π-π stacking distance drawn from the simulation of ra-P3HT was 3.98 Å, which was also close to the approximated peak value of 4.0 Å, as from the XRD measurement.

**Figure 6:**
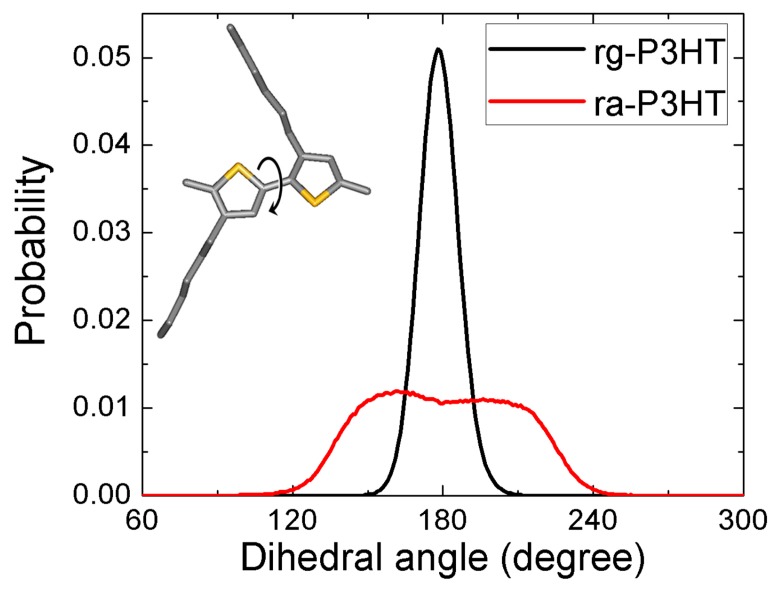
Statistical distribution of the dihedral angles between the neighboring thiophene rings in the simulated rg-P3HT and ra-P3HT chains. The dihedral angle is defined in the inset picture.

Since the carrier transport properties are closely related to the planarity of the backbone, it is important to quantitatively examine the influence of the side chain disorder on the backbone planarity. To this purpose, we calculated the distribution of dihedrals between the neighboring thiophene rings along the backbones in the simulated structures of rg-P3HT and ra-P3HT (Figure 6). As is evident from Figure 6, rg-P3HT exhibits much more planar backbones than ra-P3HT because a sharp peak at 180° appears in rg-P3HT, and the average torsion angle (dihedral deviation from 180°) is only 6 ± 5°. In contrast, the ra-P3HT film shows a much stronger deviation from planarity, as documented by a larger average torsion angle of 21° and a more dispersed angle distribution of 13°. Since the carrier transport properties were quite sensitive to the intra-chain π-π conjugation, which was dominated by the planarity of the backbone structure, we expect to obtain a much larger mobility in rg-P3HT than in ra-P3HT, based on the difference of their backbone planarity.

### 3.3. Direct Mapping of Carrier Transport Path

**Figure 7:**
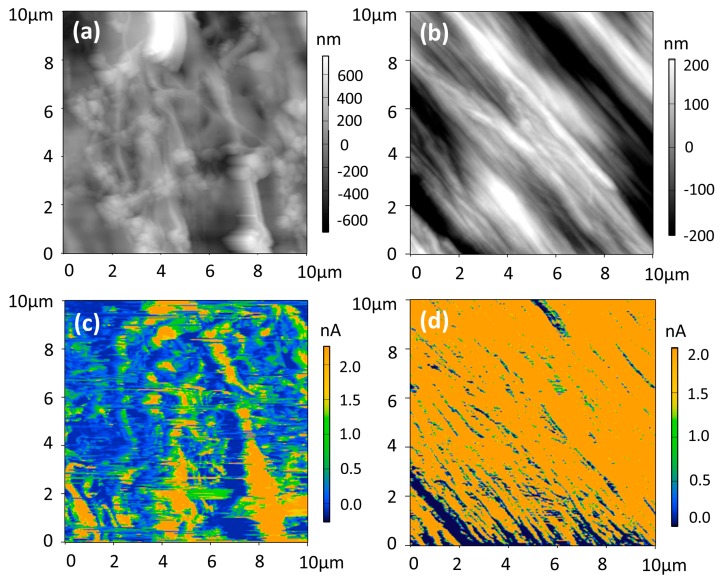
Conductive atomic force microscopy (C-AFM) mapping images. (**a**) Surface topography of ra-P3HT-TCB; (**b**) Surface topography of rg-P3HT-TCB; (**c**) Current image of ra-P3HT-TCB, and (**d**) Current image of rg-P3HT-TCB. The scanning voltages are 0.1 V.

The conductive atomic force microscopy (C-AFM) was used to map the carrier transport path in the oriented rg-P3HT-TCB and ra-P3HT-TCB films. The ra-P3HT and rg-P3HT films were also measured for comparison (as shown in Figure S3). C-AFM is a form of scanning probe technique that simultaneously measures the surface topography and the local conductivity with nanometer-scale resolution [25,26]. In C-AFM, the conducting probe makes contact with the sample (the tip acts as a nanoelectrode) and measures the current as a function of the applied voltage to map out the local current image. Figure 7 shows the surface topography and current images of the tested samples. High conductive (*I* > 1.0 nA) and low conductive (*I* < 1.0 nA) domains were clearly observed in both the ra-P3HT-TCB and rg-P3HT-TCB films, corresponding to topographically higher and lower domains, respectively. The high conductive (*I* > 1.0 nA) and topographically higher region could be classified as the domain with higher carrier delocalization, possibly due to more ordering structure. The low conductive (*I* < 1.0 nA) and topographically lower region could be classified as the domain with lower carrier delocalization, possibly due to disordering structure. Both films showed oriented fiber structures in the surface topography images, while the bundle-like structures were observed more vividly in rg-P3HT-TCB. This character was more clearly reflected in the current images, as shown in Figure 7c,d. The rg-P3HT-TCB film exhibited homogeneous bundle-like structures showing excellent high electrical conductivity along the fiber bundles (yellow regions, *I* ~ 2.0 nA). However, the current path image in ra-P3HT-TCB was characterized by a less ordered and inhomogeneous distribution. Combining C-AFM mapping and SEM observations, it was concluded that the microscopic current path features in ra-P3HT-TCB and rg-P3HT-TCB were much more different even though they had similar macroscopic structure characters of oriented dense fibers. The current path in rg-P3HT-TCB maintained the homogeneous bundle-like character even on the submicrometer length scale, while it showed the island-like character in the ra-P3HT-TCB film.

### 3.4. Carrier Transport Properties of ra-P3HT-TCB and rg-P3HT-TCB Films

**Figure 8:**
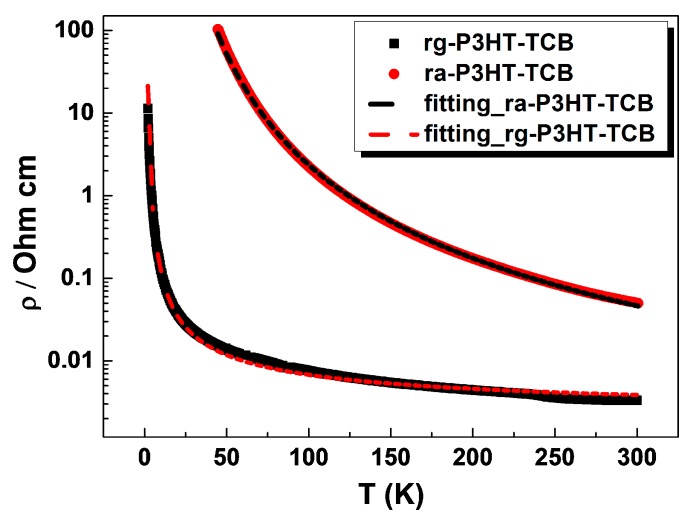
The characteristic temperature dependence of the resistivity of ra-P3HT-TCB and rg-P3HT-TCB films. The resistivity of ra-P3HT-TCB fits well with the variable range hopping conduction model, while the resistivity of rg-P3HT-TCB fits well with the quasi-1D heterogeneous hopping conduction model.

The room temperature electrical resistivity and the Seebeck coefficient of the TCB-regulated P3HT films with two different side chain RR were measured and are presented in Table 1. The TE performance of ra-P3HT and rg-P3HT are shown in Table S1. The electrical conductivity of the rg-P3HT-TCB and ra-P3HT-TCB films is 262 ± 6 S/cm and 20 ± 2 S/cm, respectively. The electrical conductivity of the former, with regular side chain RR, was more than one order of magnitude higher than that of the latter. Seebeck coefficients of ra-P3HT-TCB and rg-P3HT-TCB had similar values of 46 ± 5 μV/K and 42 ± 3 μV/K, respectively. Consequently, the power factor of rg-P3HT-TCB was as high as 39.1 ± 2.5 μW/mK^2^, exceeding the power factor of ra-P3HT-TCB (4.2 ± 0.4 μW/mK^2^) by an order of magnitude. The carrier mobility and the carrier concentration of ra-P3HT-TCB and rg-P3HT-TCB films at room temperature were also measured, and are listed in Table 1. The carrier mobility of ra-P3HT-TCB and rg-P3HT-TCB films in the direction parallel to the fiber axis were 0.2 ± 0.1 cm^2^/V·s and 2.9 ± 0.3 cm^2^/V·s, respectively. The carrier concentrations in ra-P3HT-TCB and rg-P3HT-TCB were similar with ~(4.1 ± 0.4) × 10^20^ and (4.8 ± 0.5) × 10^20^ cm^−3^, respectively. The significant enhancement in the electrical conductivity and the power factor of rg-P3HT-TCB compared to ra-P3HT-TCB was thus mainly ascribed to the increased carrier mobility. The temperature dependent resistivity of ra-P3HT-TCB and rg-P3HT-TCB films were measured and are shown in Figure 8. Generally, the temperature dependence of the conductivity in disordered polymers is believed to follow the variable range hopping (VRH) conduction model [27]:

(1) $$\rho =b\rho_{0}*\exp({\left(\frac{T_{0}}{T}\right)}^{\frac{1}{n+1}})$$

where *n* is the dimensionality of the hopping (*n* = 1, 2, 3) and *T*_0_ is the characteristic Mott temperature that generally depends on the carrier hopping barriers. In contrast, for the ordered polymers, which are typically considered to consist of a metallic region with ordered structure and an insulating region with disordered structure, the overall resistivity is usually taken as a sum of the two regions [28], i.e., as:

(2) $$\rho =a\rho_{m}*\exp\left(-\frac{T_{m}}{T}\right)+b\rho_{0}*\exp({\left(\frac{T_{0}}{T}\right)}^{\frac{1}{n+1}})$$

where *ρ*_m_ is the resistivity of metallic region, *ρ*_0_ is the resistivity of disordered regions, *T*_m_ is the energy of phonons that can backscatter carriers (taken as around 1000 K [29]), and *T*_0_ is the parameter that depends on the carrier hopping barriers, and *n* is the dimensionality of hopping (*n* = 1, 2, 3). The first contribution is related to the intrinsic quasi-1D metallic conductivity in the ordered regions, and the second term comes from the variable range hopping conduction between two ordered regions.

As shown in Figure 8, the electrical resistivity of ra-P3HT-TCB is well fitted by the VRH model (Equation (1)) with *n* = 3, suggesting that the electrical carriers in the oriented ra-P3HT-TCB film are transported by the thermally-assisted hopping of holes between states localized near the randomly distributed ‘traps’. This is probably because the large torsion angle between neighboring thiophene rings of the backbone chain of ra-P3HT increases the difficulty of electrons to delocalize, therefore causing more occupancy of the localized states. The randomly distributed localized states finally lead to a three dimensional carrier hopping in the ra-P3HT-TCB film. In contrast, the resistivity of the rg-P3HT-TCB film fits better with the heterogeneous model over the whole temperature range when *n* is taken as 1, as shown in Figure 8, which combines the quasi-1D metallic conduction with the quasi-1D hopping model. The results suggest that the two transport paths constituting the carrier transport in the rg-P3HT-TCB film are: the delocalized bandlike transport and the carrier hopping. The good fit to the quasi-1D hopping model (*n* = 1) further suggests that the hopping path is mainly along electrically isolated disordered chains as part of the conduction pathway parallel to the fiber axis. The difference of transport paths in rg-P3HT-TCB and ra-P3HT-TCB probably arises because the smaller torsion angle between neighboring thiophene rings of the backbone chain in rg-P3HT increases the electron delocalization density and favors the one-dimensional transport. Besides, as shown by the fitted values of ra-P3HT-TCB and rg-P3HT-TCB in Table 2 and Table 3, the former attains a much higher *T*_0_ value than the latter, which relates to the activation energy in the model, often referred to as the mobility edge, indicating that a lower mobility edge is needed for rg-P3HT with the high delocalization density. The results verify that the rg-P3HT-TCB film, having the regular substituted side chain structure can be activated more easily and favors one-dimensional transport along the fiber direction, and consequently resulting in the significantly improved carrier mobility compared to the unregularly substituted character of the ra-P3HT-TCB film.

## 4. Conclusions

Highly oriented P3HT films with different side chain regioregularity were prepared by combining a TCB small molecule template approach with the temperature gradient induced crystallization process. The microstructure and electrical carrier transport properties were investigated, focusing on the effect of side chain regioregularity. Utilizing molecular dynamics simulations and experimental investigations, we have demonstrated that changes in the side chain attachment to the polythiophene backbone exert a dramatic impact on both the microstructure and carrier transport properties in P3HT. Although both samples with different side chain regioregularity possess dense fiber structures, displaying highly oriented character, C-AFM mapping of the current path showed very different features. The HT side chain-attached sample (head-to-tail dyads more than 98%) showed the homogeneous bundle-like current pathway, while the HT-HH mixed chain attached sample (head-to-tail dyads about 50%) showed the island-like current pathway. The analysis of the temperature dependent electrical resistivity revealed that the high regioregularity caused a mixed conduction consisting of metallic and quasi-1D dimensional hopping paths, while the lower regioregularity led to 3D hopping transport paths. The higher regular side chain attachment results in a much more planar backbone chain and more ordered packing structure, providing a higher density of delocalized electrons along the polymer backbone chain. The presence of higher planarity of backbones not only reduces the carrier transport edge, but it also allows for one-dimensional transport in the film and thereby significantly improves the carrier mobility. The fundamental understanding of relations between molecular structure and their carrier transport properties may assist the formation of design rules for high performance OTEs of these complex systems.

## Figures and Tables

**Table 1:** Thermoelectric properties, carrier concentration and carrier mobility (hall mobility) of ra-P3HT-TCB and rg-P3HT-TCB.

Sample	Electrical Conductivity (S/cm)	Seebeck Coefficient (μV/K)	Power Factor (μW/mK^2^)	Carrier Concentration (×10^20^ cm^−3^)	Carrier Mobility (cm^2^/V·s)
ra-P3HT-TCB	20 ± 2	46 ± 5	4.2 ± 0.4	(4.1 ± 0.4)	0.2 ± 0.1
rg-P3HT-TCB	226 ± 6	42 ± 3	39.1 ± 2.5	(4.8 ± 0.5)	2.9 ± 0.3

**Table 2:** Fitted values of *aρ*_m_, *bρ*_0_, *n* and *T*_0_ in Equation (1) by using measured resistivity for ra-P3HT-TCB film; the value of *T*_m_ is taken as 1400 K [29].

	*bρ*_0_ (Ω cm)	*T*_0_ (K)	*n*
ra-P3HT-TCB	1.9 × 10^−7^	7.2 × 10^6^	3

**Table 3:** Fitted values of *aρ*_m_, *bρ*_0_, *n* and *T*_0_ in Equation (2) by using measured resistivity for rg-P3HT-TCB film; the value of *T*_m_ is taken as 1400 K [29].

	*aρ*_m_ (Ω cm)	*bρ*_0_ (Ω cm)	*T*_m_ (K)	*T*_0_ (K)	*n*
rg-P3HT-TCB	1.0 × 10^−3^	1.8 × 10^−3^	1400	177	1

## Supplementary Materials

The following are available online at http://www.mdpi.com/2073-4360/10/8/815/s1, Figure S1: UV-Vis-NIR absorption spectrum of oriented ra-P3HT and rg-P3HT before and after doping by Fe(TFSI)_3_, Figure S2: SEM images of (a) ra-P3HT and (b) rg-P3HT films. Both samples show homogeneous and compact structure, Figure S3: C-AFM mapping images. (a) Surface topography of ra-P3HT; (b) Surface topography of rg-P3HT; (c) Current image of ra-P3HT; and (d) Current image of rg-P3HT. The scanning voltages are 0.1 V, Table S1: Thermoelectric properties of self-assembly ra-P3HT and rg-P3HT, Table S2: Number Average Molecular Weight (*M*_n_), Weight Average Molecular Weight (*M*_w_) and Polydispersity Coefficient (*P*_i_) of ra-P3HT and rg-P3HT.
