# Supplementary material for: Understanding the Intrinsic Carrier Transport in Highly Oriented Poly(3-hexylthiophene): Effect of Side Chain Regioregularity
Source: Polymers (Basel). 2018 Jul 25;10(8):815. doi: 10.3390/polym10080815 (PMC6403984; doi:10.3390/polym10080815)
Supplement: Supplementary file 1 [file polymers-10-00815-s001.pdf]

Understanding the Intrinsic Carrier Transport in Highly Oriented Poly(3-hexylthiophene): Effect of Side Chain Regioregularity

Sanyin Qu, Chen Ming, Qin Yao, Wanheng Lu, Kaiyang Zeng, Wei Shi, Xun Shi, Ctirad Uher and Lidong Chen

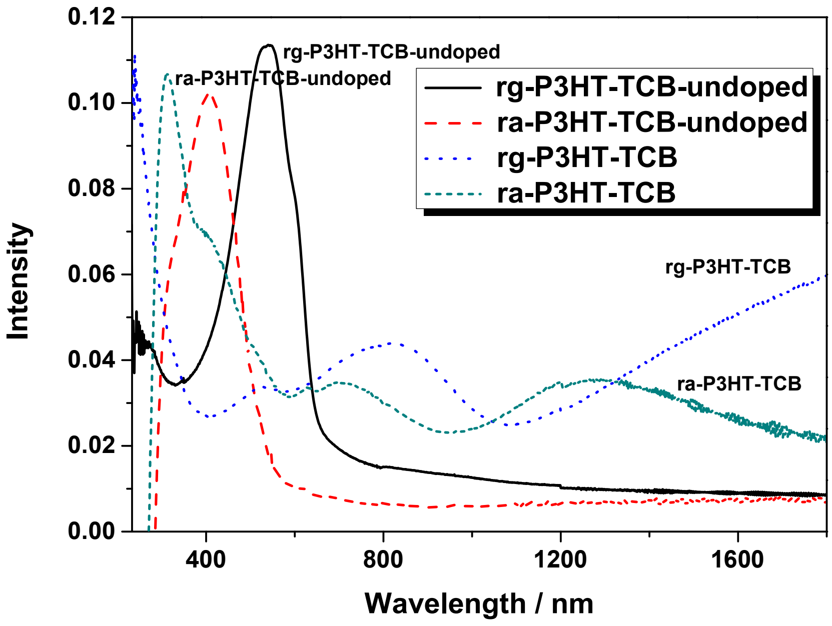

Figure S1. UV-Vis-NIR absorption spectrum of ra-P3HT and rg-P3HT before and after doping by $Fe(TFSI)_3$.

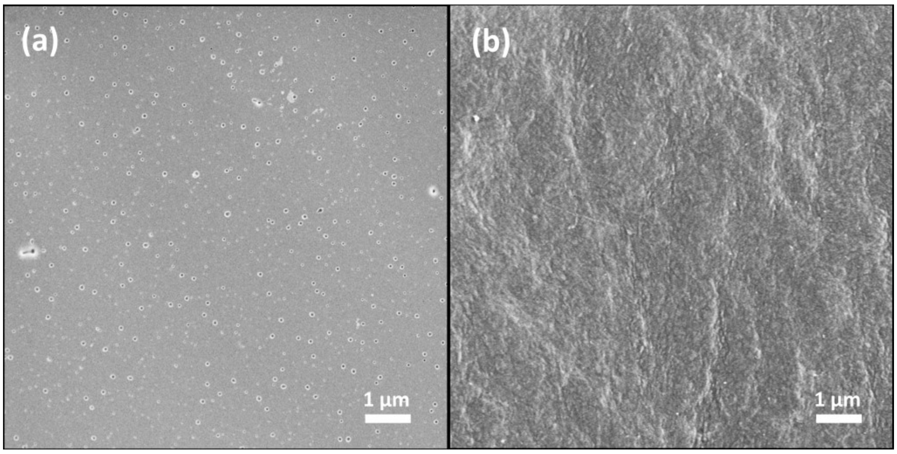

Figure S2. SEM images of (**a**) ra-P3HT and (**b**) rg-P3HT films. Both samples show homogeneous and compact structure.

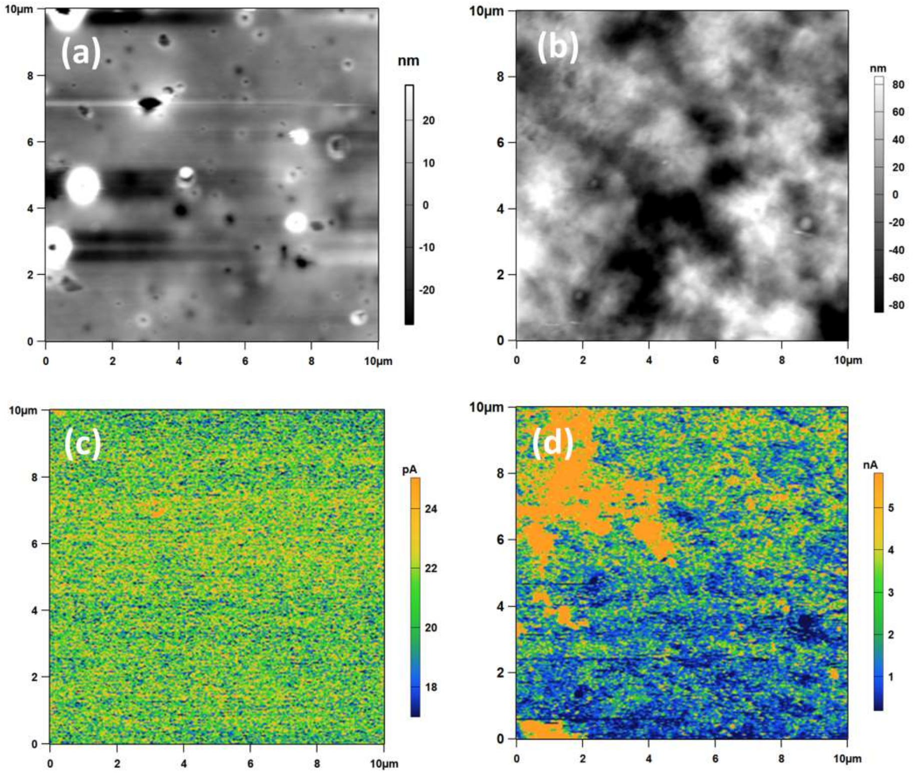

Figure S3. C-AFM mapping images. (**a**) Surface topography of ra-P3HT; (**b**) Surface topography of rg-P3HT; (**c**) Current image of ra-P3HT; and (**d**) Current image of rg-P3HT. The scanning voltages are 0.1 V.

Table S1. Thermoelectric properties of self-assembly ra-P3HT and rg-P3HT.

Sample	Electrical Conductivity (S/cm)	Seebeck Coefficient (μV/K)	Power Factor (μW/mK^2)	Carrier Concentration (× 10^{20}cm^{-3})	Carrier Mobility (cm^2/V·s)
ra-P3HT	1 ± 0.2	50 ± 5	0.25 ± 0.02	-	-
rg-P3HT	95 ± 5	40 ± 5	15.2 ± 1.1	(4.4 ± 0.6)	1.3 ± 0.2

Table S2. Number Average Molecular Weight (M_n), Weight Average Molecular Weight (M_w) and Polydispersity Coefficient (P_i) of ra-P3HT and rg-P3HT.

Material	M_n (g/mol)	M_w (g/mol)	P_i
ra-P3HT	4.26 E4	8.74 E4	2.05
rg-P3HT	4.38 E4	8.67 E4	1.98
